# Prevalence of hardcore smoking in the Netherlands between 2001 and 2012: a test of the hardening hypothesis

**DOI:** 10.1186/s12889-016-3434-x

**Published:** 2016-08-09

**Authors:** Jeroen Bommelé, Gera E. Nagelhout, Marloes Kleinjan, Tim M. Schoenmakers, Marc C. Willemsen, Dike van de Mheen

**Affiliations:** 1 IVO Addiction Research Institute, Heemraadssingel 194, 3021 DM Rotterdam, The Netherlands; 2 Erasmus Medical Center, Dr. Molewaterplein 50, 3015 CE Rotterdam, The Netherlands; 3 Department of Family Medicine (CAPHRI), Maastricht University, P.O. Box 616, 6200 MD Maastricht, The Netherlands; 4 Department of Health Promotion (CAPHRI), Maastricht University, P.O. Box 616, 6200 MD Maastricht, The Netherlands; 5 Trimbos Institute, Da Costakade 45, 3521 VS Utrecht, The Netherlands

**Keywords:** Hardcore smokers, Prevalence, Hardening, Softening, Trends

## Abstract

**Background:**

Hardcore smokers are smokers who have smoked for many years and who do not intend to quit smoking. The “hardening hypothesis” states that light smokers are more likely to quit smoking than heavy smokers (such as hardcore smokers). Therefore, the prevalence of hardcore smoking among smokers would increase over time. If this is true, the smoking population would become harder to reach with tobacco control measures. In this study we tested the hardening hypothesis.

**Methods:**

We calculated the prevalence of hardcore smoking in the Netherlands from 2001 to 2012. Smokers were ‘hardcore’ if they a) smoked every day, b) smoked on average 15 cigarettes per day or more, c) had not attempted to quit in the past 12 months, and d) had no intention to quit within 6 months. We used logistic regression models to test whether the prevalence changed over time. We also investigated whether trends differed between educational levels.

**Results:**

Among smokers, the prevalence of hardcore smoking decreased from 40.8 % in 2001 to 32.2 % in 2012. In the general population, it decreased from 12.2 to 8.2 %. Hardcore smokers were significantly lower educated than non-hardcore smokers. Among the general population, the prevalence of hardcore smoking decreased more among higher educated people than among lower educated people.

**Conclusions:**

We found no support for the hardening hypothesis in the Netherlands between 2001 and 2012. Instead, the decrease of hardcore smoking among smokers suggests a ‘softening’ of the smoking population.

## Background

In the past decades, smoking prevalence has declined globally [1], and in Western countries in particular [2]. As fewer people smoke, the remaining group of smokers may have changed over time [3].

According to the *hardening hypothesis* [3], light smokers are more receptive to tobacco control measures than heavy smokers, and they are therefore more likely to quit smoking. As the number of light smokers in the population of smokers decreases, the remaining group of smokers contains an increasingly larger portion of heavier smokers. Over time, the population of smokers would therefore become harder to reach and more difficult to change [4, 5]. In the Netherlands, for example, the prevalence of smoking decreased from 29.9 % (3.9 million people) in 2001 to 25.5 % (3.5 million people) in 2012. However, the portion of heavy smokers among those 3.9 million people in 2012, may be higher than the portion among those 3.5 million in 2001.

If the hardening hypothesis is supported, the portion of so-called ‘hardcore smokers’ in the population of smokers would have increased over the last years. Generally, hardcore smokers are smokers who have smoked for many years and do not intent to quit [4]. Compared to other smokers, such hardcore smokers are more likely to be male, to live alone and to have a lower socioeconomic status [6]. There are different definitions of hardcore smokers [7], but they generally share certain characteristics: smoking consumption, quitting history and intention to quit [6, 8–17].

On consumption, most studies agree that smokers can be classified as hardcore smokers if they smoke daily [6, 8–16] and have a minimum consumption of 15 cigarettes per day [6, 8–10, 12, 15, 16]. On quitting history and intention to quit, many of the studies on hardcore smoking only include smokers who have been smoking in the past 12 months [6, 9, 11–16] and who have no intention to quit within the next six months [8–11, 15, 16]. Finally, most studies aim to included smokers who have reached a stable smoking consumption only. They therefore limit the group of hardcore smokers to those older than 25 years [6, 8–12, 15, 16].

In this study, we chose a definition that was most similar to most of the wide variety of definitions that exist in the field. This way, the results from our study could be compared the findings of others. In addition, our criteria have been shown to be related to a lower likelihood of quitting [17]. As a result, we defined smokers as ‘hardcore’ if they were older than 25 years, smoked every day, smoked on average 15 cigarettes per day or more, had not attempted to quit in the past 12 months, and had no intention to quit within 6 months.

Previous studies suggest that smokers have hardened in some countries or within subgroups. Some found hardening among English adults from 2000 to 2010 [18] and Norwegian adolescents from 2002 to 2010 [19], Others, however, found no support for the hardening hypothesis among Norwegian adults from 1996 to 2009 [11], among Australian adults from 1997 to 2007 [20], among US adults from 1992 to 2011 [21] and among European adults from 2006 to 2012 [21].

### Educational inequalities

Educational inequalities in smoking are widening in both the Netherlands and other European countries [2, 22]. Also, not only are lower educated people more likely to be a smoker than higher educated people, they are more likely to be a *hardcore* smoker as well [6, 11]. Previous studies suggest that hardcore smoking is increasing at a higher rate among lower educated people than among higher educated people [13]. As a result, the portion of lower educated people among hardcore smokers would rise. As lower educated people are, in general, harder to reach by tobacco control messages than higher educated people [23], it would become even more difficult to affect hardcore smokers through tobacco control measures.

### Current study

The current study is performed with data from 2001 to 2012 from the Netherlands. In the current study, we investigated whether the smoking population hardened in the Netherlands during the period 2001-2012. We also investigated whether differences in hardcore smoking existed between educational levels and whether these differences have changed over time. To identify such population trends, we used repeated cross-sectional survey data from a large nationally representative sample of the general population in the Netherlands.

As smoking is predicted by education [24], the distribution of smoking across educational levels differs between the smoking population and the general population (i.e., which also includes non-smokers). The influence of education on trends in hardcore smoking among smokers may be different from that among the general population. We therefore analysed both trends in hardcore smoking among smokers and among the general population.

## Methods

### Participants

We used data from the Dutch Continuous Survey of Smoking Habits: a cross-sectional web survey that monitors the smoking habits of the Dutch population [22]. Respondents were 15 years and older, had been recruited via a market research company (TNS NIPO). They were invited to complete the questionnaire by email and all respondents have given informed consent. From 2001 until 2008, data were collected per household web interviewing, but from 2009 until 2012, data were collected per personal-level web interviewing. Between 2009 and 2012, response rates ranged from 67.5 to 70.3 % (no data is available about the response rates from before 2009). These rates are similar to those of other studies [9, 11]. After applying weights for sex, age, educational level, working hours, geographic region, urbanisation, and household size, the sample was representative for the Dutch population of 15 years and older. A more detailed description of the recruitment process and the sample characteristics can be found elsewhere [22].

The Central Committee on Research Involving Human Subjects in the Netherlands required no ethical approval for this non-medical survey research.

### Variables

#### Hardcore smoking

We categorized respondents as non-smoker, non-hardcore smoker or hardcore smoker. We determined smoking status by asking: ‘Do you ever smoke or do you not smoke at all?’ Smokers were ‘hardcore’ if they a) smoked every day [11, 14], b) smoked on average 15 cigarettes or more per day [6, 10], c) had not attempted to quit in the past 12 months [15, 16], and d) did not intend to quit within 6 months [8, 9]. All other smokers, who did not meet the criteria for being a hardcore smoker, were considered non-hardcore smokers. Comparable to previous studies, we only included participants of at least 25 years old in our analyses [8, 10]. These smokers may not have reached a stable level of average daily consumption [6]. We were unable to identify hardcore smokers in the first three months of 2001 and the last three months of 2004, due to missing values on our criterion variables. We therefore excluded participants from these periods from the analyses.

#### Respondents’ characteristics

We assessed age, sex, employment, number of cigarettes per day and whether participants used roll-your-own cigarettes or factory-made cigarettes. We assessed highest attained education and categorized participants in three groups (Dutch names in brackets). Lower educated people either received primary education, lower secondary education (MAVO) or lower vocational education (LBO). Intermediate educated people received intermediate vocational education (MBO) or higher secondary education (HAVO, VWO). Higher educated people had attained tertiary education (HBO, University).

### Analyses

First, we tested for groups differences between hardcore smokers and non-hardcore smokers on age (t-test) or any other characteristics (χ^2^-tests).

Next, we calculated the prevalence of hardcore smoking within both the smoking population and the general population. We did this for each year from 2001 until 2012. We also calculated this prevalence for each educational level separately.

Finally, we used a logistic regression model to test whether the prevalence of hardcore smoking among smokers had increased over time. This model had hardcore smoking as outcome and consisted of three steps. In the first step, we entered a dichotomous trend variable (0 for 2001, 1 for 2012). In the second step, we added a three-level ordinal variable for education. In the final step, we added interaction variables to test whether the prevalence of hardcore smoking had developed differently between educational levels. We controlled for age and sex, because age and sex are known predictors of hardcore smoking [6]. As the distribution of educational levels of the smoking population is different from that of the general population, we calculated a separate model for the prevalence of hardcore smoking among the general population.

### Secondary analysis

In a secondary analysis, we investigated whether the trend in hardcore smoking would have been different if we had used another definition of hardcore smoking. Some studies did not use consumption to define hardcore smokers [14, 25]. Therefore, in this secondary analysis we used the same regression models as described above to investigate the trend in hardcore smoking, but this time we removed our consumption criterion from our definition. As a result, in this secondary analysis, hardcore smokers were defined as those who a) smoked every day, b) had not attempted to quit in the past 12 months, and c) did not intend to quit within 6 months. Again, we only included participants of at least 25 years old in these sensitivity analyses.

## Results

### Sample characteristics

Table 1 shows the weighted distribution of sex and education in the general population from 2001 until 2012. Over the years, the weighted dataset included more males *χ*^*2*^ (1, *N* = 179371) = 4.50, *p* = .034, *φ* = .007, and higher educated participants, *χ*^*2*^ (1, *N* = 178601) = 4011.91, *p* < .001, *φ* = .189. Our weighted dataset of hardcore smokers, also included more women, *χ*^*2*^ (1, *N* = 18474) = 4.48, *p* < .034, *φ* = .031, and higher educated participants over time, *χ*^*2*^ (1, *N* = 18399) = 219.20, *p* < .001, *φ* = .169

**Table 1:** Sex and educational levels among the general population and among hardcore smokers from 2001 until 2012 (weighted data)

		2001	2002	2003	2004	2005	2006	2007	2008	2009	2010	2011	2012
General population													
Total N		11369	15536	16280	11626	16490	15370	12562	15879	16815	15992	15861	15590
Sex (%)	Male	48.8 %	48.8 %	48.8 %	48.8 %	48.8 %	48.8 %	48.8 %	48.8 %	49.5 %	49.7 %	49.2 %	49.4 %
	Female	51.2 %	51.2 %	51.2 %	51.2 %	51.2 %	51.2 %	51.2 %	51.2 %	50.5 %	50.3 %	50.8 %	50.6 %
Education (%)	Low	47.3 %	47.1 %	47.1 %	47.6 %	47.5 %	47.1 %	47.0 %	47.2 %	30.4 %	28.1 %	28.4 %	26.5 %
	Medium	31.9 %	31.9 %	31.7 %	31.2 %	31.6 %	32.1 %	32.5 %	32.4 %	40.6 %	41.0 %	39.7 %	40.0 %
	High	20.8 %	21.0 %	21.2 %	21.2 %	20.9 %	20.8 %	20.5 %	20.4 %	29.0 %	30.9 %	31.9 %	33.5 %
Hardcore smokers													
Total N		1386	1980	1926	1243	1737	1652	1335	1619	1678	1417	1219	1281
Sex (%)	Male	51.5 %	52.2 %	51.2 %	52.3 %	54.2 %	53.0 %	53.6 %	52.4 %	51.1 %	49.9 %	50.5 %	48.2 %
	Female	48.5 %	47.8 %	48.8 %	47.7 %	45.8 %	47.0 %	46.4 %	47.6 %	48.9 %	50.1 %	49.5 %	51.8 %
Education (%)	Low	57.9 %	58.0 %	58.1 %	61.2 %	62.6 %	61.0 %	60.9 %	58.9 %	45.6 %	40.2 %	41.5 %	39.9 %
	Intermediate	30.3 %	30.0 %	30.1 %	28.8 %	27.2 %	29.6 %	28.3 %	31.1 %	41.4 %	44.5 %	43.5 %	43.6 %
	High	11.8 %	12.0 %	11.8 %	10.0 %	10.2 %	9.4 %	10.8 %	10.1 %	13.0 %	15.2 %	15.0 %	16.5 %

Note: Due to missing values on criterion variables we were unable to identify hardcore smokers in the first three months of 2001 and the last three months of 2004. We therefore excluded participants from these six months from the analyses.

### Hardcore smokers vs. non-hardcore smokers

Table 2 shows the sample characteristics of both hardcore smokers and non-hardcore smokers in 2012. Compared to non-hardcore smokers, hardcore smokers were older, *t*(2873.61) = 3.23, *p* = .002, *d* = .104, and more likely to be lower or intermediate educated, *χ*^*2*^ (2, *N* = 3972) = 108.50, *p* < .001, *φ* = .165. They were also less likely to be student and more likely to be unemployed or unable to work, *χ*^*2*^ (5, *N* = 3953) = 78.23, *p* < .001, *φ* = .141. Finally, hardcore smokers were more likely than non-hardcore smokers to smoke roll-your-own cigarettes, *χ*^*2*^ (1*, N* = 3972) = 333.53, *p* < .001, *φ* = .290. We found no significant differences in sex in 2012, *χ*^*2*^ (1, *N* = 3973) = 3.78, *p* = .053, *φ* = .031.

**Table 2:** Sample characteristics of hardcore smokers and non-hardcore smokers in 2012

	Hardcore smokers (*n* = 1414)	Non-hardcore smokers (*n* = 2957)	Significance
Age (SD)^a^	49.2 (12.4)	47.9 (14.4)	*p* = .002
Sex (%)			
Male	48.2	51.5	*p* = .053
Female	51.8	48.5	
Education (%)			
Low	39.3	27.3	*p* < .001
Medium	43.6	42.4	
High	16.5	30.3	
Employment (%)			*p* < .001
Employed	56.7	67.3	
Unemployed	9.7	5.3	
Unable to work	14.0	9.0	
Retired	9.9	13.5	
Student	0.5	1.9	
Other	9.3	7.0	
Smokes RYO (%)^b^			*p* < .001
Yes	67.8	36.8	
No	32.2	63.2	

^a^For this analysis, we only included participants aged 25 years or older because hardcore smokers are by definition 25 years or older

^b^RYO: Roll-your-own cigarettes

### Prevalence

Among smokers, the prevalence decreased from 40.8 % in 2001 to 32.2 % in 2012. Among the general population, the prevalence decreased from 12.2 % in 2001 to 8.2 % in 2012. Both drops were significant, *p* < .001 (see Table 3, step 1 in both models).

**Table 3:** Logistic regressions for the prevalence of hardcore smoking

	Smokers (*N* = 7456)		General population (*N* = 27,804)	
	Adj. OR^a^	CI (95 %)	Adj. OR^a^	CI (95 %)
Step 1^a^				
*Trend*				
2001	1		1	
2012	.665***	(.603, .733)	.658***	(.606, .713)
Step 2^a^				
*Trend*				
2001	1		1	
2012	.738***	(.667, .816)	.803***	(.738, .874)
*Education*				
Low	1		1	
Intermediate	.752***	(.673, .841)	.629***	(.573, .691)
High	.424***	(.369, .489)	.305***	(.269, .346)
Step 3^b^				
Trend * Education				
Low vs. Intermediate	.903	(.725, 1.125)	.850	(.709, 1.020)
Low vs. High	.907	(.686, 1.200)	.656**	(.514, .837)

Significance: **p < .01, ***p < .001

^a^Adjusted for age and sex

^b^Adjusted for age, sex and main effects of trend and education

### Educational inequalities

**Fig. 1:**
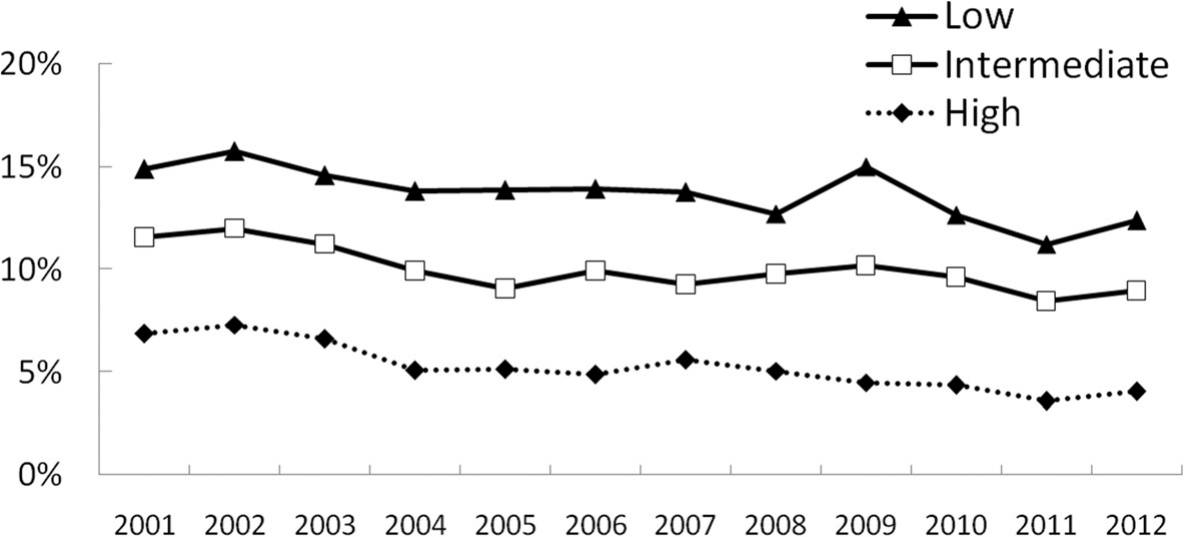
Prevalence of hardcore smoking among the general population from 2001 to 2012 by educational level (weighted data)

Step 2 in Table 3 shows the odds ratios for being a hardcore smoker for each educational level. In both populations, lower educated people were more likely to be hardcore smoker than intermediate and higher educated people. Step 3 shows the odds ratios for the interaction terms between trend and education. Among smokers, we found no trend differences between educational levels. Among the general population, however, the prevalence of hardcore smoking decreased more among higher educated people than among lower educated people, *p* < .001. The trends between lower and intermediate educated people did not differ significantly, *p* = .081. Figure 1 shows the prevalence of hardcore smoking among the general population from 2001 to 2012 for each educational level.

### Smoking consumption

The sensitivity analysis showed that removing the consumption criterion did not affect the results of any of the regression models. Among both smokers and the general population, we found a decrease in hardcore smoking over time. In both populations the trend remained significant after including education and the interaction between trend and education to the regression models.

## Discussion

### Hardening hypothesis

The hardening hypothesis predicts that the portion of hardcore smokers among smokers would increase over time. In contrast to this hypothesis, we found that among smokers the prevalence of hardcore smoking decreased from 40.8 % in 2001 to 32.2 % in 2012. In the general population, this prevalence decreased from 12.2 % in 2001 to 8.2 % in 2012. These findings suggest that, between 2001 and 2012, the Dutch smoking population has gradually softened, instead of hardened. This is in line with previous studies in Norway [11], Australia [20], and the United States [21].

The softening of the population may also be explained by a gradual decrease in the number of cigarettes smoked among smokers. As in previous studies [6, 8–10, 12, 15, 16], one criteria for hardcore smoking was smoking at least 15 cigarettes per day. As the average number of cigarettes per day smoked decreased, some hardcore smokers started smoking less than 15 cigarettes per day and may have become non-hardcore smokers over the past years. However, removing the consumption criterion from our definition of hardcore smokers, did not affect the results of our study. The softening of the population may therefore have occurred independently from the reduction in cigarette consumption.

Two other factors may also explain this softening of the smoking population. First, tobacco control policy measures, such as smoking bans and tax policies, may not only have stimulated light smokers to quit smoking, but may have influenced heavy smokers (i.e., hardcore smokers) as well. Second, the softening may be a result of changing social norms. Societal norms about smoking may have changed over time and this process might have increased quitting throughout the whole smoking population.

Both explanations are in line with Rose’s theory, which states that tobacco control measures and social norms do not only influence light smokers, but the population as a whole [26]. Therefore, the remaining group of smokers would become softer instead of harder. While tobacco control policies and changing social norms are likely causes, the decrease of hardcore smoking in the Netherlands might also have been caused by other factors, such as a higher rate of mortality among hardcore smokers than among other smokers. Following Rose’s argument, however, we expect that the prevalence of hardcore smoking continues to decline in the next years in the Netherlands. As others have found evidence of hardening in other countries [18, 19], future research may focus on the causes for hardening and softening of the smoking population to investigate why some studies found evidence for hardening, while others did not.

### Educational inequalities

In line with previous research [6, 11, 13], we found that hardcore smoking was more prevalent among lower educated people than among intermediate or higher educated people. Hardcore smoking decreased in all three groups, but we found no trend differences between educational levels among smokers. Among the general population, however, we did find such trend differences. The prevalence of hardcore smoking decreased more among higher educated people than among lower educated people. This corroborates literature on widening educational inequalities in smoking behaviour in the Netherlands [22]. The different findings between the smoking population and the general population could be explained by other trends in the Dutch general population. While the portion of higher educated people has increased in the Dutch general population [27], these higher educated people are less likely to smoke than lower educated people [22]. The general population therefore contains an increasing portion of non-smoking higher educated people over time. The smoking population, however, remains relatively unaffected by this growing group of non-smoking higher educated people. This may explain why we found trend differences between educational levels among the general population, but not among smokers.

In line with other studies [6, 12], we found that hardcore smokers were lower educated than non-hardcore smokers and that they were more likely to be unemployed. In addition, we found that hardcore smokers are much more likely to smoke roll-your-own cigarettes than non-hardcore smokers in the Netherlands. This difference may further indicate socio-economic differences, because roll-your-own cigarette smokers tend to have a lower income and to be lower educated than those who smoke factory-made cigarettes [28]. Lower costs are one of the main reasons for smoking roll-your-own cigarettes [28]. Therefore, tax policies may help to further decrease educational inequalities in hardcore smoking. Increasing tax on roll-your-own tobacco, for example, would decrease the difference in price between roll-your-own cigarettes and factory-made cigarettes.

### Strengths and limitations

A strength of our study is that we used repeated cross-sectional data from a large representative sample of the general population. This allowed us to examine trends in hardcore smoking among both smokers and among the general population. Because we had a large sample, we were also able to identify differences in trends between educational levels.

A potential concern is the definition of hardcore smokers. Although several studies investigated the prevalence of hardcore smoking before, no clear definition of hardcore smokers currently exist. In our study, we therefore used a definition that is most comparable to other studies. As many studies have used different definitions, it is difficult to compare the prevalence of hardcore smoking between studies [7]. By using a definition that is similar to others, however, we are able to compare *trends* in hardcore smoking. These trends may be more informative about future characteristics of the smoking populations than prevalence rates.

### Suggestions for future research

Future research may focus on the use of e-cigarettes among hardcore smokers. A recent study showed that Dutch tobacco smokers are increasingly aware of e-cigarettes and that many have started to use them [29]. If future population surveys do not effectively take into account e-cigarette use, this may bias future estimates of hardcore smoking. Many e-cigarette smokers have smoked traditional cigarettes before taking up e-cigarettes and remain to do so after starting to use e-cigarettes [30]. In the current study, we have assessed traditional cigarette consumption, but some smokers would be classified as hardcore nicotine users if we had assessed their e-cigarette consumption as well. Also, as e-cigarettes allow smokers to use nicotine in places where smoking traditional cigarettes is banned, smokers may be more likely to increase their total nicotine consumption and become hardcore nicotine users eventually.

### Practical implications

Despite the softening of the smoking population, about 8.2 % of the Dutch population is still a hardcore smoker. This group remains particularly vulnerable to death, disease, and lower quality of life. Therefore, interventions targeting hardcore smokers are still needed to further decrease the prevalence of hardcore smoking in the Netherlands [4]. Previous literature suggested that such interventions may incorporate motivational interviewing techniques [31] and contain targeted and tailored information [32]. Motivational interviewing aims to decrease resistance to anti-smoking messages and encourages participants to come up with arguments for behavioural change themselves. Tailored information is information that has been individualized to participants, based on, for example, their personal beliefs about smoking [32]. It has shown to increase effectiveness of web-based smoking cessation interventions [33].

In line with previous studies [6], our study showed that hardcore smoking is more prevalent among lower educated people. Interventions targeting hardcore smokers may therefore decrease educational inequalities. One such intervention encourages smoking cessation among pregnant women [34, 35]. In the Netherlands, smoking during pregnancy is particularly prevalent among lower educated people [36]. Improving interventions that encourage these hardcore smoking, pregnant women to quit smoking, may therefore not only reduce hardcore smoking, but may reduce educational inequalities in smoking as well.

## Conclusions

The prevalence of hardcore smoking among smokers *decreased* between 2001 and 2012. This suggests that the population of smokers has softened, instead of hardened. There was no support for the hardening hypothesis in the Netherlands. Among the general population, hardcore smoking decreased at a higher rate among higher educated people than among lower educated people. This may be explained by increasing educational differences in smoking among the general population.
